# Ventricular depolarisation vectors in exercise induced myocardial ischaemia

**DOI:** 10.1038/s41598-017-14865-0

**Published:** 2017-11-07

**Authors:** Cameruddin W. Vellani, Mohammad Yusuf, Sadia Mahmud, Satwat Hashmi

**Affiliations:** 1 Section of Cardiology, Department of Medicine, The Aga Khan University Stadium Road, P.O. Box 3500, Karachi, 74800 Pakistan; 2 Department of Radiology, The Aga Khan University, Stadium Road, P.O. Box 3500, Karachi, 74800 Pakistan; 3 Department of Community Health Sciences, The Aga Khan University Stadium Road, P.O. Box 3500, Karachi, 74800 Pakistan; 4 Department of Biological and Biomedical Sciences, The Aga Khan University, Stadium Road, P.O. Box 3500, Karachi, 74800 Pakistan

## Abstract

Ischaemia reduces membrane excitability and conduction of myocardial depolarisation. This would alter the synergy of electromotive forces that contribute to a resultant force at any instant. Changes in magnitude and direction of resultant forces are reflected in electrocardiographic signals. Here we show a method for obtaining the coordinates of resultant electrical forces during exercise derived from a bipolar orthogonal lead system for calculation of electrical vectors in three planes. In a trial, analysis of changes in vectors indicated that the extent of reduction in magnitude with exercise was significantly greater in groups of patients categorized by impaired effort tolerance and signs of ischaemia. Measurement of changes in the spectrum of depolarisation vectors during exercise has the potential for non-invasive assessment of myocardial ischaemia. This could be the basis of a portable, low-cost tool for investigation of patients with symptoms suggestive of coronary artery disease.

## Introduction

Transient discomfort in the chest during effort or at rest commonly leads to investigation of coronary artery disease as the cause. ST depression in the ECG during a standard exercise tolerance test (ETT) is a helpful non-invasive indicator of ischaemia; however, studies have shown considerable variation in sensitivity and specificity, with mean values of 68% and 77%, respectively^1^.

Myocardial perfusion scan (MPS) uses a gamma-camera to record reduction of the distribution of a radioactive tracer injected intravenously at the peak of exercise. Experience enables estimation of risk for acute coronary events^2^ hence its value for decisions concerning the need for angiography.

Non-invasive modalities of imaging that show high sensitivity and specificity have been developed; the value of accuracy relative to cost was assessed by Salerno and Beller^3^. In economically under-privileged societies where the prevalence of coronary disease is high^4^ these methods of investigation are inaccessible because of fixtures in few large urban centres and cost.

Experimental evidence has shown that myocardial ischaemia results in reduced myocardial excitability and conduction^5^. Variable progression of depolarisation in an ischaemic region alters the electromotive force (EMF) recorded by bipolar intra-mural electrodes, as shown experimentally^6^. Disrupted synergy of depolarisation in a wider ischaemic region should reduce its contribution to the resultant EMF at that instant and may alter its direction.

We propose that these inherent indicators provide the basis for a simpler, cost-effective alternative to MPS for diagnosis and location of myocardial ischaemia.

Vectorcardiography (VCG) derived from simultaneously recorded orthogonal lead signals, averaged to minimise artefact, enables measurement of the magnitude and direction of planar vectors during ventricular depolarisation. This paper describes the method developed at the Aga Khan University (AKU) and results of its initial trial. The study was funded by the AKU Research Council. The method has been patented (Vectorcardiographic Signal Analyser, USA. Patent No: US 9, 226, 674 B2: January 5, 2016).

## Methods

The study was carried out in accordance with relevant guidelines and regulations of the Aga Khan University and its Ethical Review Committee (ERC). All experimental protocols were approved by the Aga Khan University ERC and informed consent was obtained from all subjects.

Consistent features of the ventricular depolarisation waveform (QRS) with frequencies below 150 Hz were extracted by averaging the signals of 3 bipolar orthogonal leads recorded simultaneously. The averaged signals were largely free from artefact^7,8^ and accurately aligned with data recorded at different stages of exercise. The averaged signals provided the coordinates for measurement of the magnitudes and directions of cardiac electrical vectors in 3 orthogonal planes (horizontal, frontal, sagittal).

Electronic processing of the data enabled visual display of vectors at stages of exercise and measurement of change from the stage of reference, therefore independent of patterns of specific clinical states.

The bipolar orthogonal lead system of McFee and Parungao^9^ is suited for such measurements and was used with modification of polar orientation of the Z lead and placement of the Y+ electrode. Lead electrodes of silver-silver chloride with adhesive collars were attached relative to a point of reference (POR) with the aid of a template. The POR was the 5th intercostal space 2 cm from the left sternal border. Electrodes were located as follows:

- Z+ (anterior), 6 cm from the POR at the apex and angles of an equilateral triangle; Z− (posterior), opposite the POR.
- X+ (left), 5.5 cm vertically above and below a point aligned with the POR, one third of the anteroposterior depth of the thorax; X− (right), opposite the mid-point of the X+ electrodes.
- Y+ (inferior), 5 cm below and left of the umbilicus, vertically aligned with the POR; Y− (superior), anterior angle of the left posterior triangle of the neck.
- Ground, 5 cm below and right of the umbilicus.

Resistors were connected between electrodes and lead terminals: Z+ , 100 K Ohm on each electrode; X+ , 66 K Ohm on each electrode. No resistors were connected to Z−, X−, Y + , Y− and Ground electrodes.

The virtual intersection of the 3 lead axes was estimated to lie in the cavity of the left ventricle approximately 25% of the distance from apex to the aortic valve.

Lead signals below 150 Hz were amplified by 3 isolated Coulbourn V75-4 bio-amplifiers. Electrical equipment in the test room was grounded through the amplifiers.The amplified lead potentials were digitised at intervals of 1.5 milliseconds (ms) in supine, sitting and standing positions before treadmill exercise and stored. Lead signals were recorded at each stage of the Bruce protocol^10^ to provide 30–80 QRS complexes. The digital data were processed by software developed at AKU.

The baseline before each QRS was defined by the average voltage over 12–21 ms, excluding the downstroke of P at peak exercise.

### Identification of main features of the ECG signal

The first derivative of the ECG signal was obtained and smoothed to minimise noise. The beginning and end of the QRS waveform were marked by the first derivative and enabled identification of the QRS by means of various rule sets. Of the first derivative, the upstroke of R is marked by a positive peak, the peak of R by its transition at zero, and the down stroke of R by its negative trough. The R peak in one lead (usually X) was designated the reference called the “centre point” (CP) for data of the 3 lead signals.

A fixed interval before and after the CP marked the window of analysis.

### Process of averaging the QRS waveform

The QRS waveform was separated from noise by averaging accurately aligned data points.

In each lead signal consecutive waveforms that appeared to be consistent were selected as the initial reference for averaging the data of successive waveforms in the window of analysis.

Accurate alignment of the initial selected QRS waveforms in the window of analysis was obtained by cross-correlation of data of the second waveform with the first, beginning with an offset of a certain number of data points. The sum of products of the corresponding data points in this position was calculated. The sample signal was then shifted by one point and the sum of products calculated; this process was repeated a certain number of times.

The correspondence of data points that gave the largest sum of products indicated the best match of the sample signal with the reference. The data points of the reference and sample signal were averaged at this optimal correspondence. Significant variance in the highest sum of products excluded a waveform different from the usual.

**Figure 1:**
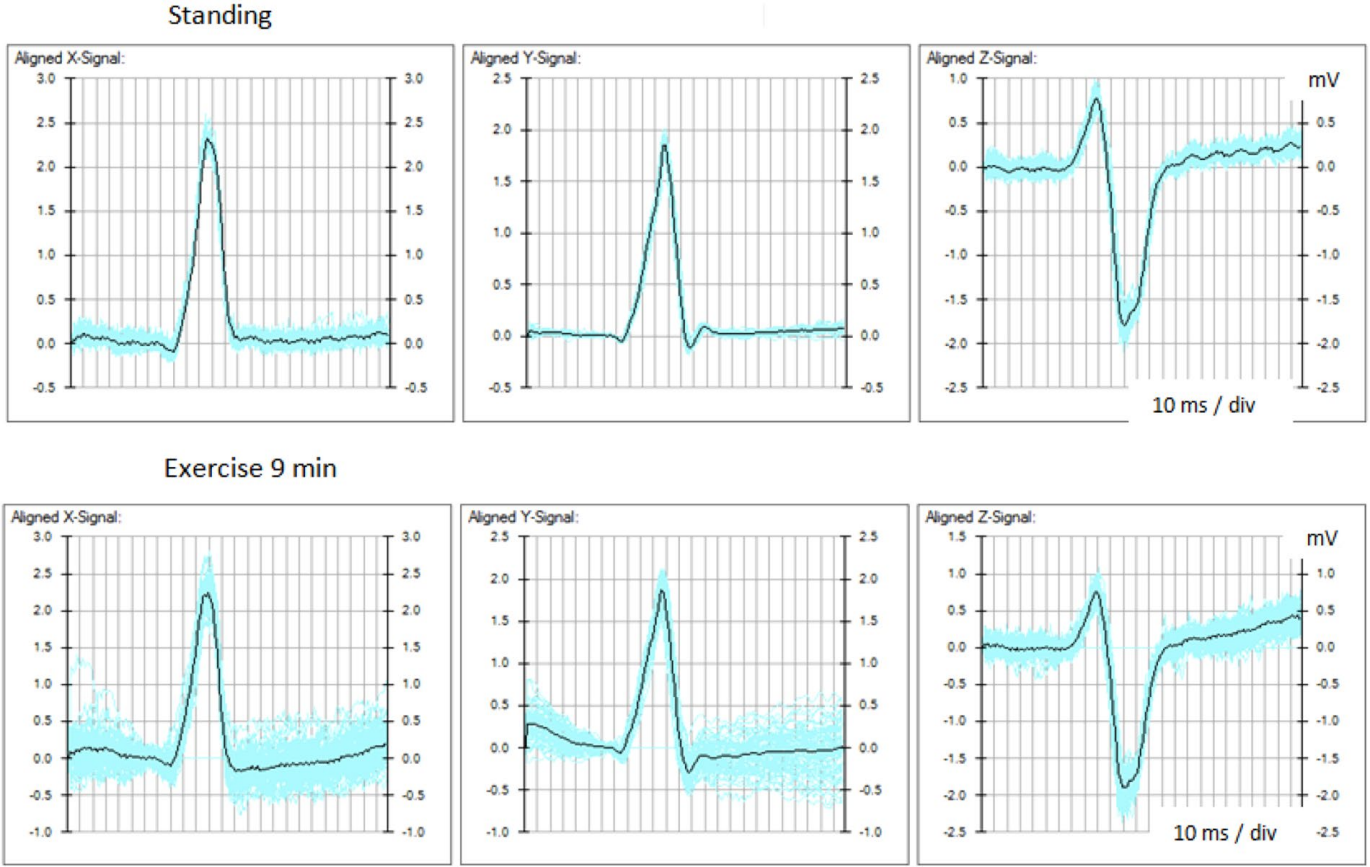
Averaged QRS signal. The continuous black line shows the baseline adjusted, averaged QRS waveform extracted from the variations of the signal (blue) due to electromyographic and other electrical potentials recorded before and at the peak of exercise.

The averaged signal in turn became the reference for cross-correlation with data of the next waveform. The final result of the baseline adjusted, averaged QRS complex, with variations in values of the data points is shown in Fig. 1. The averaged lead signals represented consistent characteristics of ventricular depolarisation of the respective stages of the exercise test.

**Figure 2:**
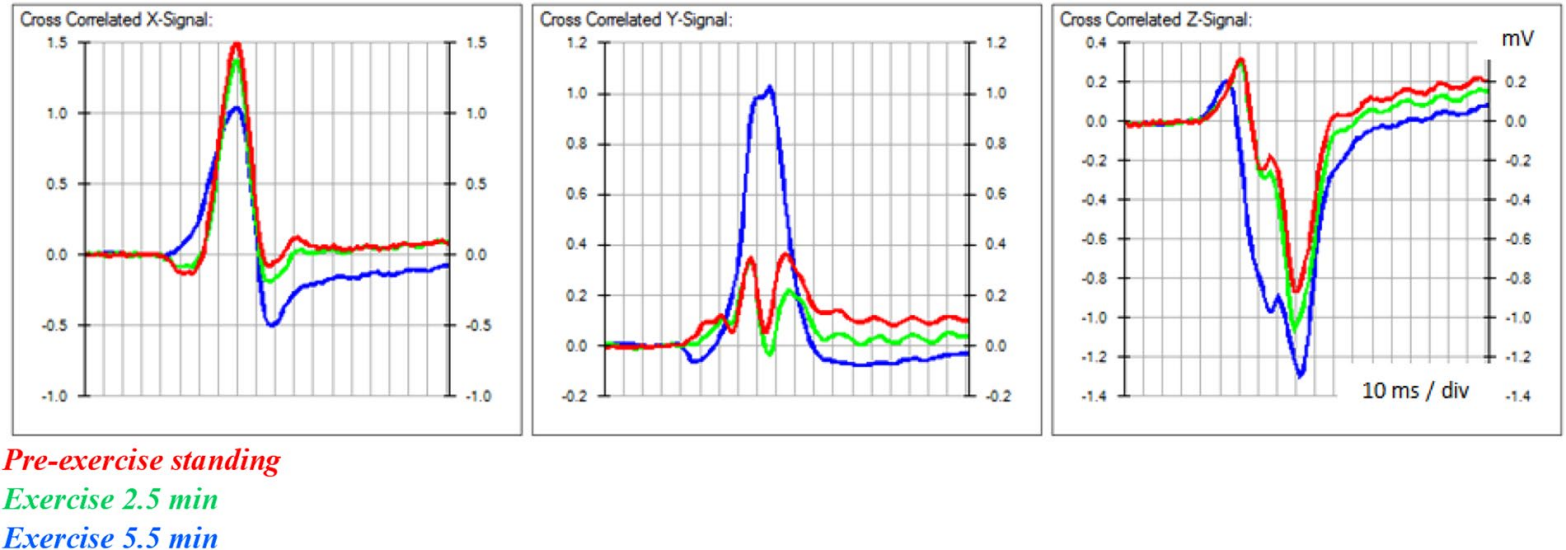
Alignment of QRS waveforms. The averaged QRS waveforms before and during exercise are accurately aligned for measurement of changes in the magnitude and direction of vectors derived from lead signals.

An example of accurate alignment of waveforms during exercise despite marked change in the sequence of depolarisation is illustrated in Fig. 2.

The averaged data of the orthogonal leads at any stage of the exercise test represented the signals of that stage. The data points of the leads provided the coordinates for calculation of the planar magnitudes and angles of the vectors and enabled measurement of the changes in planar vectors at any stage relative to the magnitudes and directions at the stage of reference just before exercise. Display of vectors at various stages of the exercise test provided visual assessment of changes, as shown in the Results section. The numerical values and their timings were tabulated for analysis of the extent and location of changes by algorithms in *Excel*.

### Initial trial of the method

#### Subjects

Male patients of the Aga Khan University Hospital, without bundle branch block or recent myocardial infarction, referred for assessment of ischaemia as the cause of symptoms by exercise and MPS, were studied after informed consent. The mean age was 52.18 (SD ± 12.40) years. Data were recorded also from asymptomatic male volunteers aged 22–30 (median 23, IQR 6) years who did not smoke, exercised regularly and consented to a standard ETT, without cost.

Subjects were excluded from study if signal artefacts compromised computation of one lead. Records of 31 patients and 15 volunteers were substantially complete and studied in groups categorised by symptoms and signs of ischaemia and tolerance of exercise, as shown in Table 1.

**Table 1:** Characteristics of subjects studied for changes in depolarisation vectors.

Groups	n	Exercise (minutes)	Chest pain or ST change on ETT	MPS reversible perfusion defect
A	14	>8.5	No	0
B	4	<8.5	No	0
C	5	>8.5	Yes	0
D	8	<8.5 (7 patients) 9 min (1 patient)	Yes	8
V	15	>10	No	Not done

Groups A–D refer to patients and Group V to the volunteers.

The study was carried out in accordance with relevant guidelines and regulations of the Aga Khan University and its Ethical Review Committee (ERC). All experimental protocols were approved by the Aga Khan University ERC and informed consent was obtained from all subjects.

### Representation of changes in magnitude and direction of vectors

Change in vector magnitude was considered as change in the proportion of the averaged magnitudes of 3 planar vectors relative to the corresponding averaged values of the reference state. A proportional change of 0.1 indicated a change of 10% and was represented by a score of one unit, referred to as relative vector magnitude (RVM). Differences greater than 5 units were recorded but not considered because indicators of ischaemia were being sought and changes in the magnitude and direction of vectors greater than 50% were due to marked alteration in the sequence of depolarisation. The use of scores had the advantage of accommodating extraneous variations in the lead signals that might have affected the averaged QRS waveform.

Change in direction of a spatial vector was represented by a score derived from the mean of the absolute differences of its 3 planar angles from the corresponding angles before exercise. A score of 1 unit indicated a mean difference of 10°; differences greater than 50° were considered to be indicators of changes in the sequence of depolarisation, not ischaemia.

### Statistical analysis

Changes in vectors during exercise, represented by scores, were analysed in the groups of patients (A-D) and the group of volunteers (V) categorised in Table 1.

Of the total of 31 patients, 2 of Group D were unusual hence excluded from analysis; in one, RVM was grossly enhanced, the second was the only one of the group who exercised longer than others but there were no data for the earlier stage at 5.5 minutes. Thus data for a total of 44 subjects were available for statistical analysis; of these 29 were patients and 15 were volunteers.

For each stage of the exercise test the scores of enhanced and reduced RVM, and change in angle scores were summed separately over the depolarisation cycle. Each outcome was analyzed for differences between exercise stages and groups by fitting a mixed model using PROC MIXED in SAS (*SAS 9.1.3. Cary, NC: SAS Institute Inc; 2000*) with group as the between-subject factor and exercise stage as the within-subject factor. For each of the three models autoregressive AR(1) covariance structure was optimal based on AIC and BIC criteria^11^. For multiple comparisons Bonferroni and Tukey-Kramer adjustment were used (*SAS 9.1. 3 Help and Documentation. Cary, NC: SAS Institute Inc; 2000*).

## Results

### Examples of changes in vectors

**Figure 3:**
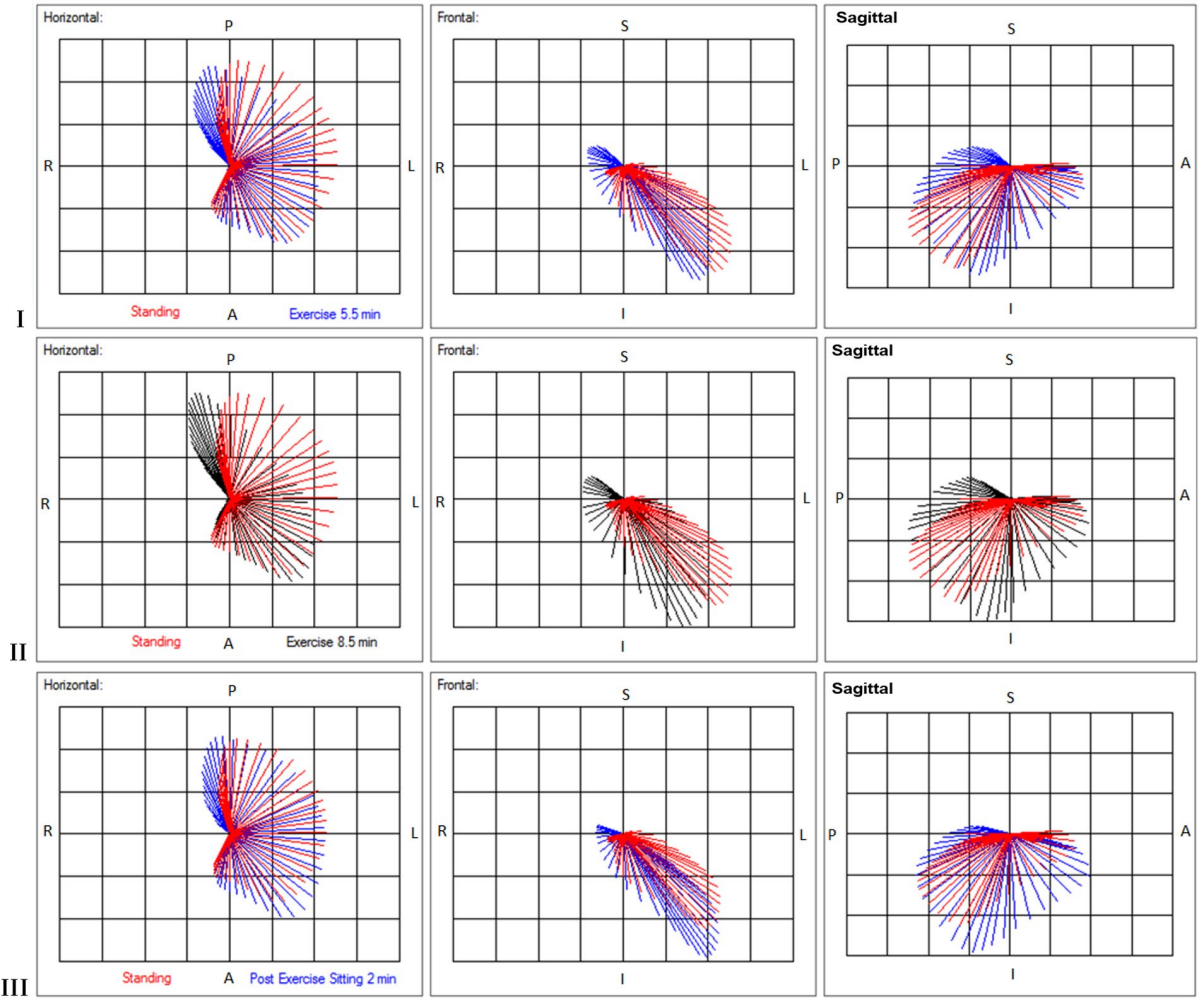
Display of magnitudes and direction of planar vectors. Reduction in the magnitudes of vectors directed posterior-left, left-inferior and posterior-inferior shown in horizontal, frontal and right sagittal planes, respectively, occurs at 5.5 min and further at 8.5 min of exercise and is largely resolved at 2 min post exercise. Inferior vectors are enhanced, posterior vectors are displaced rightward and superior. Orientation of the vectors: A, anterior; P, posterior; L, left; R, right; S, superior; I, inferior.

Figure 3 shows progressive changes in the magnitude and direction of vectors during exercise and early resolution of these changes 2 min post exercise, consistent with ischaemia induced by physical effort but effort tolerance and MPS were normal. The cause of the changes is indeterminate but could be due to changes in the sequence of depolarisation.

**Figure 4:**
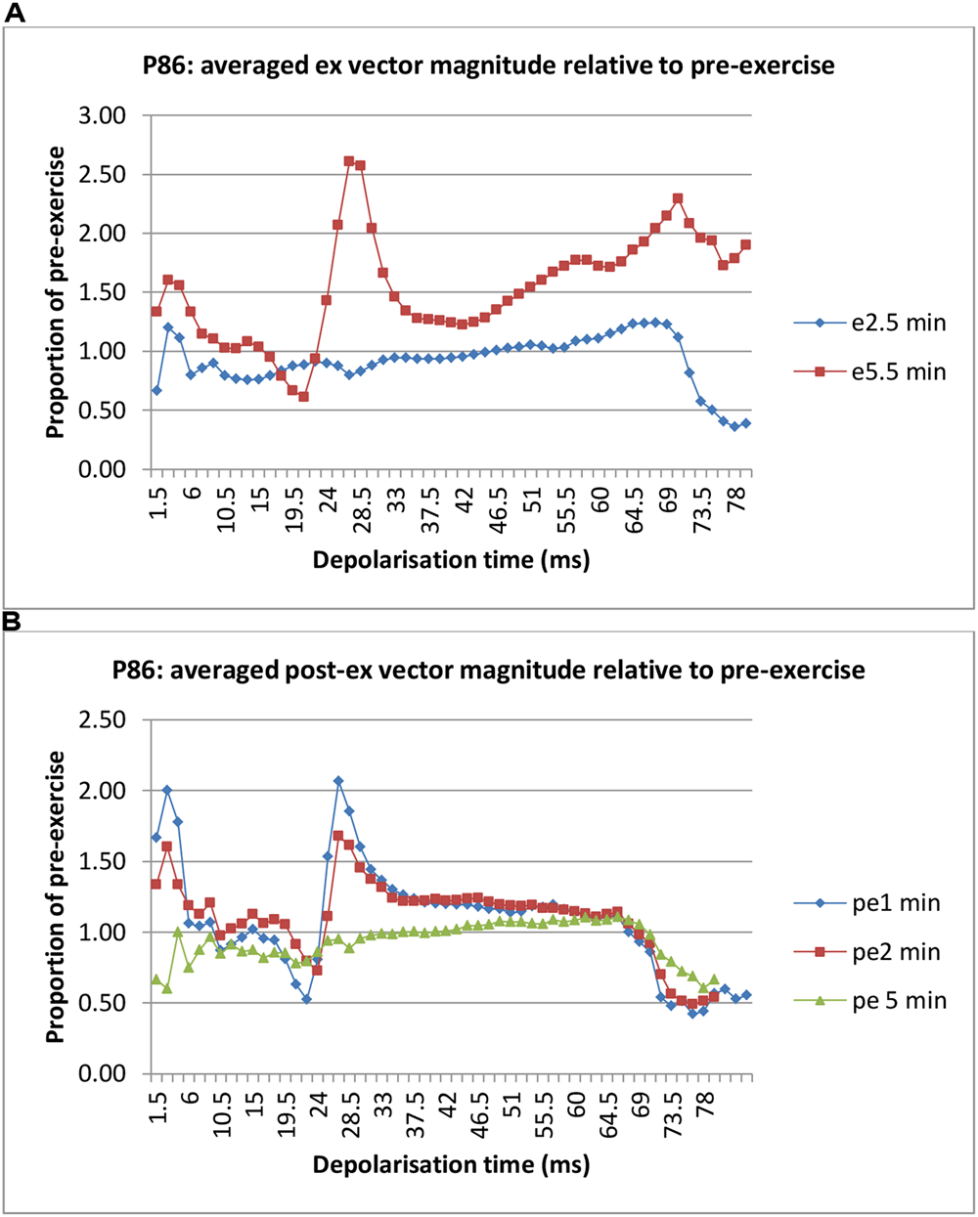
Changes in vector magnitude in the course of ventricular depolarisation. Panel A shows marked enhancement of RVM at 5.5 min of exercise occurring abruptly at 22.5 ms of depolarisation overriding the trend of reduction. Panel B shows resolution at 5 min post exercise (pe). The references for RVM during and after exercise were the lead signals while standing pre-exercise and sitting post-exercise, respectively.

Figure 4 shows reduction and subsequent abrupt enhancement in RVM indicating alteration in the sequence of regional depolarisation, consistent with failure in the conduction pathway to the region. The abrupt enhancement occurring early in ventricular depolarisation and subsequent resolution over 5 min indicate that the change is probably due to ischaemia affecting the sub-endocardial Purkinje network. MPS in this patient showed reversible inferior-apical and anteroseptal perfusion defects. Coronary angiography 3 days later showed proximal LAD 100% occlusion, with retrograde distal filling, and 99% stenosis of distal RCA.

### Reduced RVM scores

Mixed model analysis with the above outcome indicated that there was a marginally significant interaction between factors group and exercise stage (p-value = 0.0783).

For volunteers and patients of group A there was no significant change in the mean reduced RVM scores during exercise up to 8.5 minutes (Table 2). Significant reduction of the mean scores of reduced RVM was shown at 5.5 minutes of exercise for patients in group B and marginally significant for group D. Significant reduction was shown at 8.5 minutes of exercise in patients of group C, the mean scores at 2.5 and 5.5 minutes being similar.

**Table 2:** Effect of group and exercise stage on mean^†^ (standard error) reduced RVM scores.

Exercise stage	Group A	Group B	Group C	Group D	Group V
2.5 min	−25.6 (5.2)	−26.8 (9.7)	−20.8 (8.6)	−34.5 (7.9)	−24.5 (5.0)
5.5 min	−30.6 (5.2)	−41.3 (9.7) (p-value = 0.0359)	−20.6 (8.9)	−44.0 (7.9) (p-value = 0.0903)	−26.1 (5.0)
8.5 min	−35.2 (5.4) (p-value = 0.2074)	—	−42.8 (8.6) (p-value = 0.0034)	—	−27.5 (5.0) (p-value = 0.8033)

^†^Least square means are reported. Significant reduction in relative vector magnitude (RVM) scores at 5.5 minutes of exercise in groups B and D and at 8.5 minutes in Group C indicates induction of ischaemia at different levels of physical work hence severity of compromised myocardial perfusion.

### Enhanced RVM scores

Mixed model analysis with the above outcome indicated that there was a marginally significant interaction between factors group and exercise stage (p-value = 0.1074). Significant increment of the mean scores of enhanced RVM was shown at 5.5 minutes of exercise in groups B and D (Table 3). Significant increment was shown at 8.5 minutes of exercise in volunteers and patients in group A, but not in group C.

**Table 3:** Effect of group and exercise stage on mean^†^ (standard error) enhanced RVM scores.

Exercise stage	Group A	Group B	Group C	Group D	Group V
2.5 min	39.5 (7.6)	43.75 (14.2)	57.8 (12.7)	50.7 (11.6)	44.1 (7.3)
5.5 min	55.2 (7.6)	67.25 (14.2) (p-value = 0.0036)	61.1 (12.8)	81.7 (11.6) (p-value < 0.0001)	57.3 (7.3)
8.5 min	63.6 (7.8) (p-value = 0.0014)	—	64.2 (12.7) (p-value = 0.8602)	—	69.4 (7.3) (p-value = 0.0016)

^†^Least square means are reported. Enhancement of the relative vector magnitude (RVM) occurs in the second half of the depolarization cycle. Significant enhancement of the RVM scores occurs at a lower level of physical work in patients of Groups B and D who also had significant reduction in RVM consistent with ischaemia. Enhancement of RVM is a manifestation of change in the sequence of depolarization.

### Change-in-angle scores

There was a highly significant effect of exercise stage on the change-in-angle scores (p-value < 0.0001) adjusting for groups; mean change in angle scores at 2.5 min, 5.5 min and 8.5 min were 44.9, 63.8 and 79.0, respectively. Multiple comparisons indicated that all three means at the three stages of exercise were significantly different. Mixed model analysis showed no significant difference in the mean scores among the five groups (p-value = 0.1629) after adjusting for exercise stage. Hence changes are exercise stage dependent but not among the groups.

### Inference of the results of changes in vectors

Changes in magnitude and direction of vectors were found in patients and volunteers.

Analysis showed that the mean scores of reduced RVM were reduced significantly at 5.5 minutes of exercise in patients of Group B and with marginal significance in Group D. The effort tolerance was limited in groups B and D but defects in perfusion and chest pain or ST depression occurred only in Group D. Patients of Group C achieved their target heart rates, had chest pain or ST depression and significant reduction of the mean reduced RVM score at 8.5 minutes but did not have perfusion defects on MPS.

The extent of reduction in vector magnitude relative to work might identify subjects with coronary insufficiency that may or may not present with suggestive symptoms or signs. The stochastic nature of scores of change in one parameter related to physical work precluded definition of discriminatory criteria that could be applied in individual subjects. Understanding of the causes of change in vectors in healthy subjects is needed before criteria of ischaemia as the cause can be defined.

### Location of ischaemia

Spatial location of ischaemia was deduced from the initial direction of vectors that were subsequently reduced in magnitude during exercise, with reference to the sequence of depolarisation described by Durrer *et al*.^12^, and inferred from the direction of displacement of altered vectors. In patients of Group D, the resultant multiple pointers included regions of reduced perfusion shown by MPS. Assessment of congruence with locations identified by MPS requires definitive indicators of ischaemia.

## Discussion

Early studies of VCG and ECG compared the sites of myocardial infarction with their locations at autopsy and indicated potentially better correlation of VCG than ECG but less specificity^13^. McNeill *et al*.^14^ noted that VCG indicated infarction earlier than the ECG.

In subjects with and without coronary artery disease shown by angiography, Kilpatrick^15^ found no advantage of VCG over ECG during exercise for detection of ischaemia by changes towards established patterns of infarction in horizontal and frontal planes.

Continuous monitoring of the VCG has been used essentially to study the prognostic significance of changes in ST vectors in patients admitted for acute coronary events^16,17^.

The possibility of identifying ischaemia during ventricular depolarisation was explored by Toledo *et al*.^18^; they found reduction in high frequency components of ventricular depolarisation in the ECG to be more sensitive and specific than conventional ST analysis in detecting exercise-induced ischaemia shown by MPS.

Our study was based on reduction of excitability and conduction of depolarisation of myocardial and Purkinje tissue caused by ischaemia, conditions that would result in reduced synergy of EMF within a region hence its contribution to the resultant vector at that time, as indicated by experiment^5,6^. Therefore reversible alteration in the magnitude of the resultant vector, with or without change in direction, at specific times of ventricular depolarisation was a potentially measureable marker of ischaemia.

Our study has demonstrated the ability to measure small changes in the magnitude and direction of resultant electrical vectors at intervals of 1.5 ms through ventricular depolarisation during exercise. This was possible only by averaging the data of 30–80 QRS waveforms in order to extract consistent components of the waveform without distortion of signal frequencies and magnitudes induced by concomitant EMG and filtration. The result was a set of data that represented the vectors at a stage of exercise and enabled comparison of accurately aligned data of other stages in the same subject.

This crucial ability revealed, with high resolution, changes in the magnitude and direction of vectors that were unexpectedly similar in the records of patients and healthy volunteers yet different in extent and stage of exercise at which the changes occurred. Statistical pointers of difference in reduction of vector magnitude summed through depolarisation encouraged further study with detailed analysis of new data in order to define specific differences that could indicate myocardial ischaemia reliably. New data have been obtained and are being analysed. Statistically significant differences between vectors of patients and healthy volunteers are evident but definition of criteria of ischaemia applicable to an individual subject’s performance is difficult. Nevertheless, this method of analysis has the potential for understanding the electrophysiological basis of changes in vectors during exercise in healthy volunteers that could lead to definition of differences induced by relatively brief periods of ischaemia. Analysis of the second set of data and its implications will be presented for publication.

### Data availability statement

The datasets generated during and/or analysed during the current study are available from the corresponding author on reasonable request.
